# Targeting leukemic stem and progenitor cells expressing different BCR::ABL1 levels: antileukemic activity of asciminib with or without TKIs

**DOI:** 10.3389/fphar.2026.1780054

**Published:** 2026-03-23

**Authors:** Michele Massimino, Elena Tirrò, Chiara Romano, Stefania Stella, Cristina Tomarchio, Silvia Rita Vitale, Livia Manzella, Francesco Di Raimondo, Fabio Stagno, Paolo Vigneri

**Affiliations:** 1 Department of General Surgery and Medical-Surgical Specialties, University of Catania, Catania, Italy; 2 Center of Experimental Oncology and Hematology, A.O.U. Policlinico “G. Rodolico - S. Marco”, Catania, Italy; 3 Department of Medical and Surgical Sciences and Advanced Technologies “G.F. Ingrassia,” Anatomic Pathology, University of Catania, Catania, Italy; 4 Department of Clinical and Experimental Medicine, University of Catania, Catania, Italy; 5 Department of Precision Medicine in Medical, Surgical and Critical Care (Me.Pre.C.C.), University of Palermo, Palermo, Italy; 6 Division of Hematology, A.O.U. Policlinico “G. Rodolico - S. Marco”, Catania, Italy; 7 Hematology Section, A.O.U. Policlinico “G. Martino”, University of Messina, Messina, Italy; 8 Division of Oncology, Humanitas Istituto Clinico Catanese, Catania, Italy

**Keywords:** asciminib, BCR::ABL1, leukemic stem cells, LTC-ICs, TKIs

## Abstract

Tyrosine kinase inhibitors (TKIs) targeting ABL1 catalytic activity have markedly improved Chronic Myeloid Leukemia (CML) outcomes, inducing unprecedented and durable therapeutic responses. However, while TKIs efficiently target committed leukemic progenitors, they fail to eradicate leukemic stem cells (LSCs), which may drive disease relapse. High BCR::ABL1 transcripts at diagnosis confer a proliferative and survival advantage and are associated with a higher risk of CML progression to the acute phase. Specifically Targeting the ABL Myristoyl Pocket (STAMP) compounds, including asciminib (ASC), provide a novel mechanism to inhibit BCR::ABL1 catalytic activity. ASC is FDA-approved for patients who have failed one or more TKIs, and its efficacy has been evaluated as monotherapy, and in combination with different TKIs, in T315I-positive or advanced-phase CML. We investigated the cytotoxic effects of ASC, alone or with imatinib (IM) or nilotinib (NIL), on committed progenitors and LSCs from CML patients expressing high or low BCR::ABL1 at diagnosis. ASC reduced BCR::ABL1-dependent survival and impaired clonogenicity in committed progenitors with low, but not high, BCR::ABL1 transcripts. ASC also disrupted LSC self-renewal, reducing both frequency and number of Long-Term Culture-Initiating Cells (LTC-ICs). When combined with IM or NIL, ASC restored TKI activity against LTC-ICs expressing high BCR::ABL1 transcripts, with the association of ASC and NIL reducing both LTC-IC division rates and LTC-IC-derived CFUs. These findings suggest that ASC, alone or with NIL, may target LSCs and improve outcomes in patients with high BCR::ABL1 expression at diagnosis.

## 1 Introduction

Chronic Myeloid Leukemia (CML) is a hematopoietic stem cell disorder driven by the BCR::ABL1 oncogene, which arises from a reciprocal translocation between chromosomes 9 and 22, generating the Philadelphia (Ph) chromosome (Stagno et al., 2010). BCR::ABL1 encodes for a constitutively active non-receptor tyrosine kinase, promoting the clonal expansion of leukemic stem cells (LSCs) (Pierro et al., 2025). First-line treatment for CML relies on ABL1-targeted tyrosine kinase inhibitors (TKIs), which usually achieve impressive hematologic, cytogenetic, and molecular responses (Stagno et al., 2025). Despite these results, some patients develop resistance to TKIs, and only 15%–20% attain the deep molecular responses required for treatment discontinuation (Pierro et al., 2025). Furthermore, approximately 50% of patients who discontinue TKIs experience relapse, reflecting the persistence of LSCs that are refractory to ABL kinase inhibition (Campiotti et al., 2017). While TKIs efficiently suppress the proliferation of committed Ph + progenitors, they fail to eradicate LSCs, which retain self-renewal capacity and can generate additional malignant clones, contributing to disease progression (Braun et al., 2020). Elevated BCR::ABL1 transcripts have been associated with reduced TKI efficacy and increased risk of blast crisis (Barnes et al., 2005; Modi et al., 2007; Vigneri et al., 2017; Massimino et al., 2023). Recent molecular docking approaches have identified a class of small molecules that inhibit ABL1 kinase activity without interacting with the ATP-binding site. These STAMP compounds act as negative allosteric modulators by docking into the myristoyl pocket and stabilizing an inactive conformation of ABL1 (Fallacara et al., 2014; Malik et al., 2021). Among them, Asciminib (ASC) was the first FDA-approved STAMP inhibitor (Schoepfer et al., 2018), demonstrating significant therapeutic efficacy in both newly diagnosed patients (Hochhaus et al., 2024). and those displaying the T315I mutation or failing one or more TKIs (Rea et al., 2021; Yeung et al., 2022). Since ASC and TKIs bind distinct regions of ABL1, they can potentially be combined, and preclinical studies indicate that dual ABL1 targeting can overcome TKI resistance, reduce CML proliferation, and eradicate xenografts without recurrence (Wylie et al., 2017; HJ and Friedman, 2020; Hall et al., 2021). Indeed, multiple clinical trials are currently assessing ASC in various Ph-positive patient populations (NCT04795427, NCT04925479, NCT05143840, NCT04948333, NCT04877522). In this study, we investigated how BCR::ABL1 transcripts at diagnosis influence ASC antileukemic activity. We isolated committed progenitor cells and LSCs from patients with either high or low BCR::ABL1 expression at diagnosis and evaluated ASC as a single agent or in combination with imatinib (IM) and nilotinib (NIL). ASC reduced BCR::ABL1 kinase activity and impaired clonogenicity in committed progenitors with low BCR::ABL1 levels, but its effect was diminished in cells with high oncogene transcripts. Notably, ASC inhibited LSC self-renewal irrespective of BCR::ABL1 expression and restored TKI sensitivity in cells with high oncogene levels, without inducing cytotoxicity in healthy cells.

## 2 Methods

### 2.1 Patients

Chronic phase CML patients, selected from previously published cohort (Massimino et al., 2023), were stratified based on their *BCR::ABL1/GUS* ^*IS*^ (GUS: glucuronidase-β, IS: International Standard Scale) levels at diagnosis into low (<10%, n = 8) and high (>20%, n = 9) groups, as determined by real-time PCR according to an internationally-established protocol (Vigneri et al., 2017). All patients were followed in the Division of Hematology, A.O.U. Policlinico - S. Marco, and provided written informed consent for the use of their samples for research purposes. Individual anonymity was preserved in accordance with the Declaration of Helsinki. The study was conducted under a protocol approved by the Local Ethics Committee Catania 1, A.O.U. Policlinico-Vittorio Emanuele (34/2013/VE).

### 2.2 CD34-positive cell isolation

Bone marrow (BM) mononuclear cells (MNCs) from CML patients at diagnosis, were isolated by density-gradient centrifugation using Ficoll-Paque Premium (GE Healthcare) according to the manufacturer’s protocol and cryopreserved in freezing medium [Fetal Bovine Serum (FBS from Euroclone) supplemented with 10% dimethyl sulfoxide (Sigma)]. For all experiments, cryopreserved MNCs were rapidly thawed at 37 °C, resuspended in 50 mL of pre-chilled Iscove’s Modified Dulbecco’s Medium (IMDM) (Sigma-Aldrich) with 10% FBS (thawing medium) and centrifuged at 300 *g* for 10 min at 4 °C. Cells were then incubated with 100 μg/mL DNaseI (Stem Cell Technologies) in Hank’s balanced salt solution (HBSS) for 15 min at room temperature, washed with thawing medium and subjected to CD34-positive magnetic selection employing the CD34 MicroBead kit (Miltenyi Biotec) as previously described (Massimino et al., 2021a; Massimino et al., 2021b). The purity of the final cell population was verified using a flow cytometer with anti-CD34-PE (Beckman Coulter) and anti-CD45-FITC (Beckman Coulter) antibodies resulting in >90% purity. CD34-positive cells derived from CML patients expressing low and high BCR::ABL1/GUSIS levels at diagnosis were designed as CD34BALow and CD34BAHigh, respectively. CD34-positive progenitors isolated from healthy donors (CD34H) (n = 3) were purchased from American Type Culture Collection (ATCC).

### 2.3 Drug treatments and cell culture

Healthy and leukemic CD34-positive cells (300x103/mL) were exposed to the indicated plasma concentration of ASC (1.2 µM) (Saunders et al., 2016), IM (2 µM), NIL (2 µM) (Bradeen et al., 2006) (all from Novartis) or with their combination for 24 h in StemSpan Serum-Free Expansion medium (SFEM, Stem Cell Technologies). For the colony-forming units (CFUs) assay, the SFEM medium was supplemented with the StemSpan Cytokine Cocktail containing recombinant human (rh) Flt-3 ligand, rh stem cell factor (100 ng/mL), rh IL-3 and rh IL-6 (20 ng/mL). For long-term culture initiating cell (LTC-IC) assays, we employed 5 ng/mL for rh Flt-3 ligand and rh stem cell factor, and 1 ng/mL for rh IL-3 and rh IL-6 (Massimino et al., 2021b). At this time, cells were used to perform both CFU and LTC-IC assays.

### 2.4 Colony-forming units (CFUs) and long-term culture initiating cell (LTC-IC) assays

For CFUs assays, 500 cells were implanted in methylcellulose (Methocult H4435, StemCell Technologies) containing the above-specified drug concentrations. Colony counting was performed after 14 days according to the manufacturer’s instructions (StemCell Technologies) (Massimino et al., 2025). Briefly, for total CFUs (tCFUs), enumeration was performed counting all colonies regardless of their differentiation status. CFU - erythroid (CFU-E) and burst-forming unit - erythroid (BFU-E) were identified and counted according to their hemoglobinized status defined by a red or brown color. CFU - granulocyte macrophage (CFU-GM), were distinguished by both larger diameter and the absence of a hemoglobinized status. The LTC-IC assay was used to measure four self-renewal properties, namely: LTC-IC frequency, number, division rate and LTC-IC-derived CFUs. LTC-IC frequency was measured with the Limiting Dilution Analysis (LDA) as follows. 1.5 × 104 M2-10B4 mouse fibroblast (Stem Cell Technologies) were established as feeder layers in 96-well plates and blocked for 24 h with 2 μg/mL Mitomycin C (Sigma Aldrich). Treated and untreated leukemic and healthy CD34-positive cells were plated on the M2-10B4 (StemCell Technologyes) feeder in long-term culture medium (MyeloCult H5100 from StemCell Technologyes) for 5 weeks with weekly half-medium changes. After 5 weeks, cells were overlaid with methylcellulose (Methocult H4435, StemCell Technologies) supplemented with conditioned medium derived from 5637 cells (DSMZ) (Konig et al., 2008; Konig et al., 2008). Colonies were counted under the microscope after two additional weeks. LTC-IC frequency was calculated using the L-Calc software (StemCell Technologies) (Sutherland et al., 1990; Liu et al., 2013). LTC-IC-derived CFUs were measured performing LTC-IC assays bulk analysis. Briefly, 10x103 leukemic and healthy CD34-positive test cells were grown on 445 × 103 M2-10B4 feeder cells, treated as described above, in a 35 mm dish. After 5 weeks, adherent and non-adherent cells were collected and 5 × 104 leukemic or 10 × 103 healthy cells were resuspended in methylcellulose (H4435) as previously reported (Heaney et al., 2010; Liu et al., 2013). LTC-IC-derived CFUs were expressed as the number of clonogenic progenitors obtained after 15 days in methylcellulose (number of colonies multiplied by the total number of hematopoietic cells counted after 5 weeks of culture) divided by the number of initial test cells seeded on fibroblasts. The LTC-IC number was obtained calculating the ratio between the number of initial cells used for the bulk analysis and the LTC-IC frequency value extrapolated from the LDA analysis. The LTC-IC division rate was defined as the ratio between the LTC-IC-derived CFUs and the total number of LTC-ICs (Liu et al., 2013).

### 2.5 In-cell Western (ICW) assay for pCRKL level measurement

For the ICW assay, 20 × 10^3^ leukemic CD34-positive cells were plated in 96-well plates (previously coated with a 100 μg/mL poly-L-lysine solution) and either left untreated or exposed to ASC (1.2 µM), IM (2 µM), NIL (2 µM) or their combination for 24 h in StemSpan Serum-Free Expansion medium (SFEM, Stem Cell Technologies). Cells were fixed adding 37% formaldehyde directly into each well (final concentration 4% per well) for 20 min at room temperature with gentle rotation, permeabilized three times for 5 min with 0.1% Triton X-100 in PBS (100µL/well) and blocked with LI-COR Odyssey blocking buffer (LI-COR Biosciences) for 1 h at room temperature. Cells were then incubated with a mixture of antibodies containing monoclonal anti-CRKL (clone A1, sc-365471, Santa Cruz) and polyclonal anti-phospho-CRKLY207 (3181S, Cell Signaling Technologies) at 4 °C overnight. Cells were then washed five times for 5 min with PBS containing 0.1% Tween-20 and incubated with secondary antibodies IRDye 680RD goat anti-mouse (P/N 926-68070, LI-COR Biosciences) and IRDye 800CW goat anti-rabbit (P/N 32211, LI-COR Biosciences) diluted in Odyssey blocking buffer. After a 1-h incubation, cells were washed five times (5 min each) with PBS containing 0.1% Tween-20 and the plate was then scanned using the Odyssey Fc Imaging System (LI-COR Bioscience). All values obtained from primary antibody treatment were background subtracted from wells treated only with a secondary antibody. Relative quantification of both total and phospho-protein expression was normalized using the CellTag 700 Stain (LI-COR Bioscience). Data were analyzed using the Image Studio Software (LI-COR Biosciences).

### 2.6 Statistical analysis

Paired t-tests were performed using GraphPad Prism 8.0 (GraphPad Software Inc).

## 3 Results

### 3.1 ASC reduces the clonogenic potential of committed CD34BALow but not CD34BAHigh cells

**FIGURE 1:**
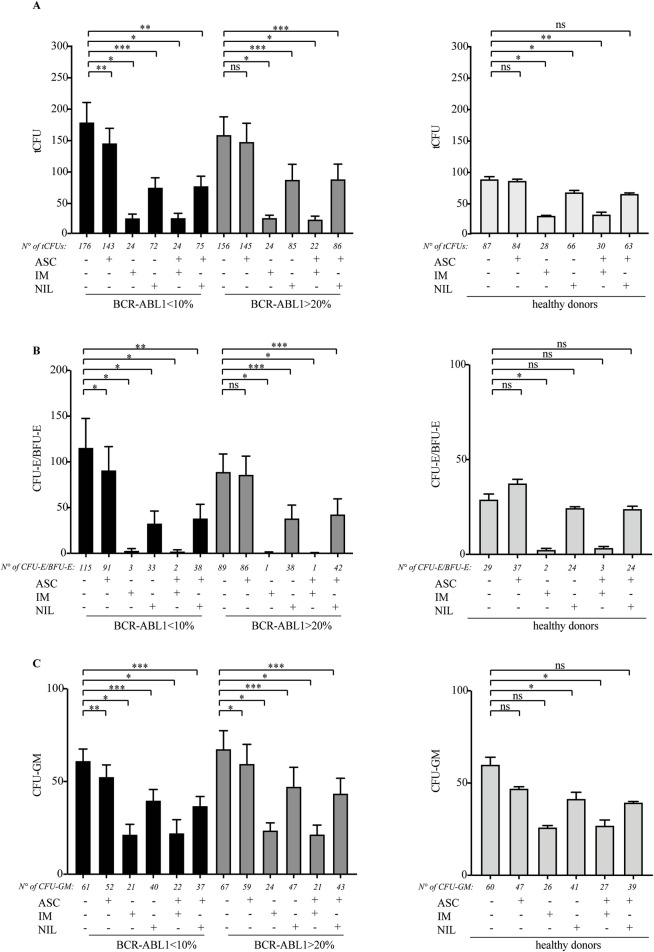
ASC reduces the clonogenic potential of CD34-positive leukemic cells expressing low or high BCR::ABL1/GUSIS levels but does not enhance TKIs activity. **(A–C)** Methylcellulose assays performed on CD34-positive cells derived from CML patients expressing low (<10%, black columns; n = 8) or high (>20%, grey columns; n = 9) BCR::ABL1/GUSIS levels (left panels), and from healthy donors (right panels; n = 2). Progenitor cells were exposed for 24 h to ASC (1.2 µM), imatinib (IM, 2 µM), nilotinib (NIL, 2 µM), or their combinations prior to plating. Histograms show the number of total CFUs **(A)**, CFU-E/BFU-E **(B)**, and CFU-GM **(C)** counted after 14 days of culture. Bars represent the mean ± standard error of the mean (SEM) from experiments performed in triplicates for each patient. Statistical significance was determined using paired t-tests (ns: not significant, *p < 0.05, **p < 0.01, ***p < 0.001). CFU: Colony-Forming Unit; CFU-E: Colony-Forming Unit - erythroid; BFU-E: Burst-Forming Uni - Erythroid; CFU-GM: Colony-forming Unit - Granulocyte Macrophage.

To determine the anti-clonogenic effect of ASC on CD34BALow and CD34BAHigh cells, we performed CFU assays detecting tCFU (Figure 1A), CFU-E/BFU-E (Figure 1B) and CFU-GM (Figure 1C). Compared to the untreated condition, ASC was more effective on CD34BALow than on CD34BAHigh, while IM and NIL showed a more potent effect on total colony reduction. In combination with either TKI, ASC did not further enhance clonogenic reduction, possibly because of the high toxic effect already exerted by IM or NIL monotherapy (Figure 1A, left panel). We next investigated the effect of ASC on the subpopulation of committed leukemic cells by counting CFU-E/BFU-E and CFU-GM. For CFU-E/BFU-E, ASC reduced colony numbers in CD34BALow cells but was ineffective against CD34BAHigh when compared to untreated cells. As expected, IM and NIL induced robust decrements in CFU-E/BFU-E regardless of their BCR::ABL1 expression (Bartolovic et al., 2004; Belle et al., 2012). This reduction was not enhanced when ASC was combined with the TKIs, suggesting that the final effect should be mostly attributed to TKI exposure (Figure 1B, left panel). When we evaluated CFU-GMs, we found that the allosteric inhibitor significantly reduced colony formation independently of their BCR::ABL1 transcripts. However, IM and NIL induced a stronger reduction in colony formation that was not increased by the combination of ASC, again indicating that the decrease in CFU-GMs was largely dependent on TKI cytotoxicity (Figure 1C, left panel). Lastly, ASC - alone or in combination with IM or NIL - displayed modest toxicity on healthy CD34+ cells (CD34H) (Figures 1A–C, right panels).

### 3.2 ASC reduces CRKLY207 phosphorylation levels in committed progenitors

**FIGURE 2:**
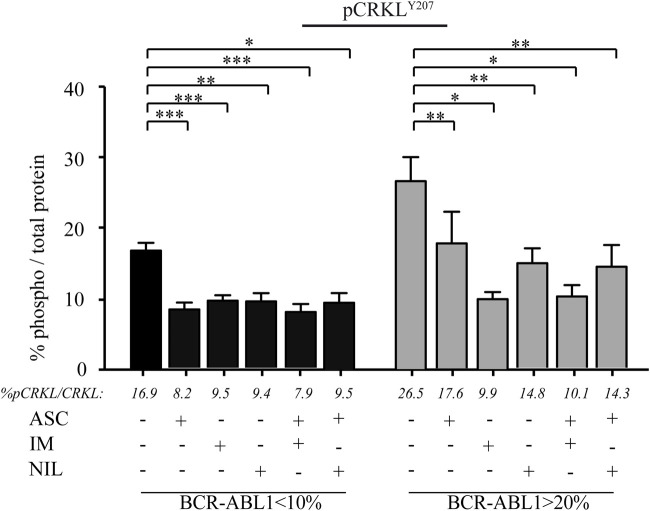
ASC reduces CRKL phosphorylation in CD34-positive leukemic cells expressing low or high BCR::ABL1/GUSIS levels. In-cell Western analysis performed on CD34-positive leukemic cells from CML patients expressing low (<10%, black columns; n = 7) or high (>20%, grey columns; n = 7) BCR::ABL1/GUSIS. Cells were treated for 24 h with ASC (1.2 µM), IM (2 µM), NIL (2 µM), or their combinations. Histograms represent phosphorylated CRKL levels normalized to total CRKL expression and expressed relative to untreated controls (set to 100%). Bars represent mean ± SEM from duplicate experiments for each patient. Statistical significance was assessed using paired t-tests (ns: not significant, *p < 0.05, **p < 0.01, ***p < 0.001).

The SH2-SH3 adapter protein CRKL is one of the major substrates of BCR::ABL1 catalytic activity with the oncoprotein directly phosphorylating its tyrosine residue in position 207 (CRKLY207). Thus, we measured the phosphorylation levels of CRKLY207 as an indirect surrogate marker of BCR::ABL1 kinase activity (Konig et al., 2008). We assessed CRKLY207 phosphorylation in CD34BALow and CD34BAHigh cells and found that ASC reduced CRKLY207 phosphorylation with lower efficacy in clones displaying higher BCR::ABL1 transcripts. No additive effect was generated by the combination of ASC with IM or NIL, regardless of the amount of BCR::ABL1 mRNA levels (Figure 2).

### 3.3 ASC interferes with the self-renewal of LSCs

**FIGURE 3:**
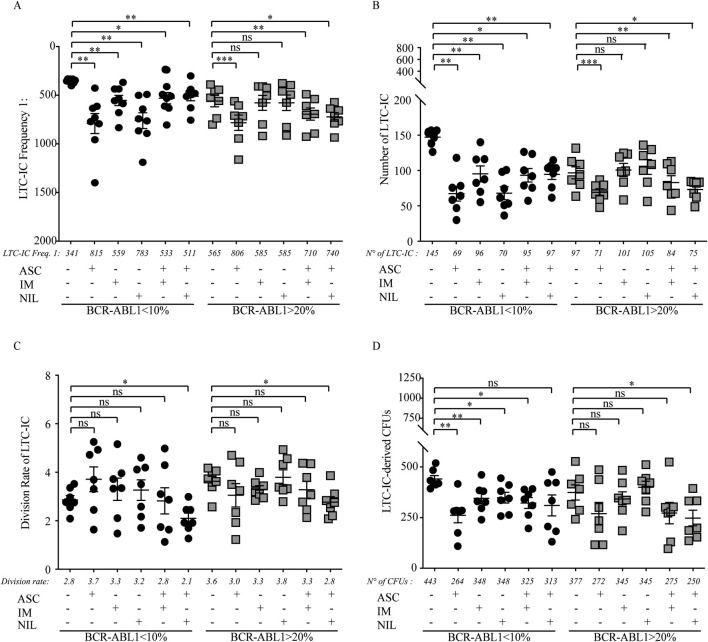
ASC affects the self-renewal properties of LTC-IC expressing low and high BCR::ABL1 levels. **(A–D)** Long-term culture-initiating cells (LTC-ICs) were obtained from CML patients expressing low (<10%, black circles; n = 8 for A, n = 7 for **(B–D)** or high (>20%, grey squares; n = 7) BCR::ABL1/GUSIS levels. Dot plots show the frequency **(A)**, total number **(B)**, division rate **(C)**, and CFU output **(D)** of LTC-ICs following 24-h exposure to ASC (1.2 µM), IM (2 µM), NIL (2 µM), or their combinations. Bars represent mean ± SEM from all experiments performed for each patient. Statistical significance was calculated using paired t-tests (ns: not significant, *p < 0.05, **p < 0.01, ***p < 0.001).

LSCs are a subpopulation of primitive leukemic cells capable of self-renewal, maintaining an undifferentiated state and generating a myeloid progeny for at least 5 weeks in long-term culture–initiating cell (LTC-IC) assays (Liu et al., 2013). We performed an LTC-IC assay to assess four parameters of LSC self-renewal: LTC-IC frequency, absolute cell number, division rate, and clonogenic potential (CFU generation) and performed statical comparison with untreated condition. ASC, IM, and NIL, either alone or in combination, showed no significant toxicity toward healthy LTC-ICs (Supplementary Figures S1A–D). However, ASC reduced LTC-IC frequency more efficiently than IM or NIL in cells expressing low or high BCR::ABL1. Unexpectedly, the addition of IM or NIL attenuated the effect in CD34BALow, while we detected a further reduction in LTC-IC frequency in CD34BAHigh cells (Figure 3A). We then quantified the absolute number of LTC-ICs and found that ASC reduced their number regardless of their BCR::ABL1 expression, although the compound was more effective in the CD34BAHigh population. Finally, addition of IM or NIL to ASC generated no benefit when compared to ASC alone (Figure 3B). We next investigated if ASC could modulate LTC-IC division rates and found no differences among the various treatment conditions except for the ASC + NIL combination, which reduced the division rate of both CD34BALow and CD34BAHigh (Figure 3C). When we examined LSC clonogenic potential, we found that ASC was more effective than IM and NIL on CD34BALow cells but failed to generate an additive effect in combination with either of the two TKIs. Remarkably, the combination of ASC + NIL (but not ASC + IM) successfully reduced LTC-IC-derived CFUs in the CD34BAHigh population (Figure 3D). Additional statistical comparation between treatment conditions was also performed (Supplementary_Tables_t-test).

## 4 Discussion

In this study, we investigated the antileukemic activity of the BCR::ABL1 allosteric inhibitor asciminib (ASC), alone or combined with imatinib (IM) or nilotinib (NIL), in leukemic cells from CML patients with low or high BCR::ABL1 transcript levels at diagnosis. Our results show that although ASC was less potent than TKIs in reducing the clonogenic potential of committed progenitors, it was more effective in impairing leukemic stem cell (LSC) self-renewal. CFU assays revealed that ASC preferentially reduced colony formation in CD34BALow cells, whereas combined allosteric and kinase inhibition reduced colonies in both CD34BALow and CD34BAHigh populations, to an extent comparable to TKIs alone, indicating no substantial enhancement of TKI-mediated cytotoxicity.

These findings partially differ from those reported by Eide et al. (Eide et al., 2019), who observed greater CFU reduction with ASC + NIL compared with NIL alone. However, their experiments were conducted on mononuclear cells rather than sorted CD34+ progenitors with distinct BCR::ABL1 expression levels. Furthermore, they used did not evaluate ASC as a single agent and used a higher ASC concentration (5 µM) than our study (1.2 µM) (Saunders et al., 2016).

CRKL phosphorylation at tyrosine 207, an indirect surrogate marker of BCR::ABL1 activity (Konig et al., 2008), was reduced by ASC in a BCR::ABL1-dependent manner, with stronger inhibition in CD34BALow cells. This contrasts with studies performed in unsorted mononuclear cells and underscores the importance of analyzing defined progenitor populations to accurately assess ASC activity (Eide et al., 2019).

Notably, ASC was more effective than IM or NIL in reducing LTC-IC frequency regardless BCR::ABL1 expression at diagnosis and restored TKI sensitivity in LTC-ICs with high BCR::ABL1, highlighting its ability to target LSC self-renewal. These results align with previous evidence showing that TKIs fail to eradicate LSCs (Corbin et al., 2011; Hamilton et al., 2012). Interestingly, IM and NIL partially attenuated ASC-mediated LTC-IC reduction, particularly in CD34BALow cells, suggesting that the efficacy of ASC and TKI combinations may depend on BCR::ABL1 levels and inhibitor stoichiometry. Structural studies have demonstrated that ASC and NIL can co-bind a single BCR::ABL1 molecule, and *in vitro* experiments suggest additive effects of ASC-TKI combinations (Wylie et al., 2017). Our data indicate that the efficacy of ASC in combination with TKIs may be dependent on the relative levels of BCR::ABL1, implying that the stoichiometric ratio between the oncoprotein and the inhibitors could critically influence the outcome of dual-targeting strategies.

LTC-IC division rates (He et al., 2009) showed that only ASC + NIL reduced stem cell division in both CD34BALow and CD34BAHigh populations, likely reflecting the greater kinase inhibitory potency of NIL. ASC alone impaired clonogenicity in LTC-ICs with low BCR::ABL1, while effects in high BCR::ABL1 cells required combination with NIL.

Recent clinical studies support ASC as a first-line therapy in newly diagnosed CML, demonstrating superior efficacy over investigator-selected TKIs (Hochhaus et al., 2024), with additional benefit reported for ASC alone or in combination regimens (Rea and Hughes, 2022). Our data provide mechanistic support for these findings, indicating that ASC efficacy may stem from its ability to target LSC self-renewal. Moreover, additional studies evaluating ASC efficacy in Ph + ALL have reported positive effects on leukemic stem/progenitor cells, further reinforcing our findings and supporting the potential role of ASC as an effective strategy for targeting and possibly eradicating *BCR::ABL1*-positive stem cell populations (Chatain et al., 2024; Chanut et al., 2025). Finally, our results also show a favorable toxicity profile, as ASC exerted limited effects on healthy cells in our experimental models. Consistent with our findings, clinical studies have also reported an acceptable safety profile in ASC-treated patients even if a potential risk of cross-toxicity when combined with TKIs cannot be excluded (Perez-Lamas et al., 2023), hence, the apparent discrepancy between preclinical safety and observed clinical toxicity warrants further clarification.

Given that treatment-free remission (TFR) represents a major therapeutic goal in CML (Shah, 2019; Hochhaus et al., 2020) and that molecular relapse remains frequent due to persistent LSCs (Etienne et al., 2017; Radich et al., 2021; Rea et al., 2018; Hochhaus et al., 2020), strategies aimed at LSC eradication are essential (Houshmand et al., 2019; Massimino et al., 2021b; Soverini et al., 2021). Overall, our findings support ASC, alone or combined with NIL, as a promising approach to eliminate LSCs, deepen molecular responses, and promote durable TFR. Unlike TKIs, ASC directly targets LSC maintenance and may modulate the leukemic stem cell niche, positioning it as a key agent in achieving sustained remission, including in patients with high BCR::ABL1 transcript levels at diagnosis (Ernst et al., 2024; Yoshimaru and Minami, 2024).

## Data Availability

The original contributions presented in the study are included in the article/Supplementary Material, further inquiries can be directed to the corresponding author.

## Supplementary material

SUPPLEMENTARY FIGURE S1 Lack of toxicity of ASC, alone or combined with TKIs, in LTC-ICs derived from healthy donors. **(A–D)** LTC-IC assays performed on healthy donors (n = 3). Dot plots show LTC-IC frequency **(A)**, total number **(B)**, division rate **(C)**, and CFU output **(D)** following a 24 h exposure to ASC (1.2 µM), IM (2 µM), NIL (2 µM), or their combinations. Bars represent mean ± SEM from all experiments performed for each donor. Statistical significance was assessed using paired t-tests (ns: not significant).
