# Supplementary figures and images for: Targeting leukemic stem and progenitor cells expressing different BCR::ABL1 levels: antileukemic activity of asciminib with or without TKIs
Source: Front Pharmacol. 2026 Mar 23;17:1780054. doi: 10.3389/fphar.2026.1780054 (PMC13051198; doi:10.3389/fphar.2026.1780054)

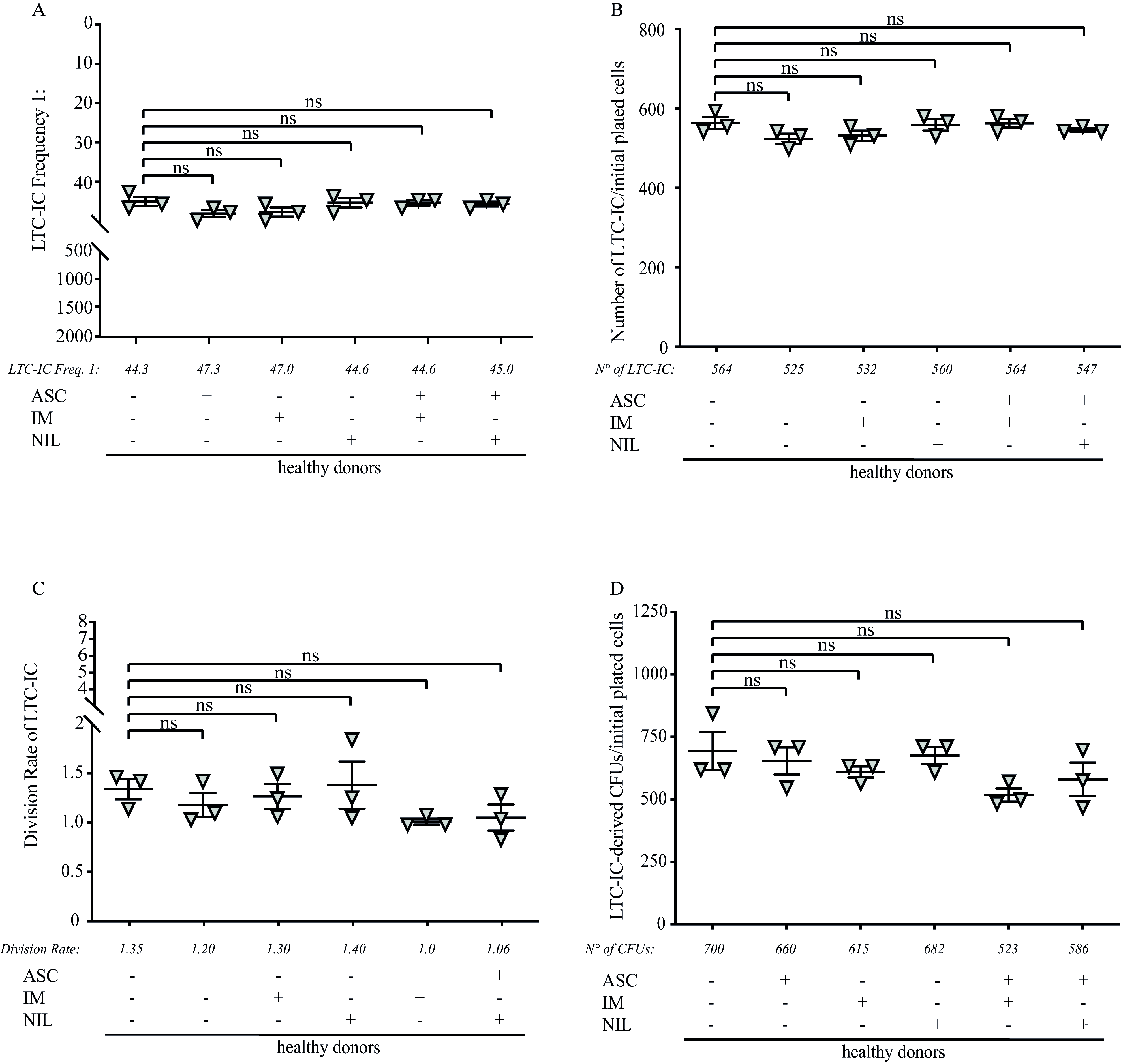

Supplement: Supplementary file 1 [file Image1.tif]
